# Discriminating between Parallel, Anti-Parallel and Hybrid G-Quadruplexes: Mechanistic Details on Their Binding to Small Molecules

**DOI:** 10.3390/molecules27134165

**Published:** 2022-06-29

**Authors:** Tarita Biver

**Affiliations:** Department of Chemistry and Industrial Chemistry, University of Pisa, Via G. Moruzzi 13, 56124 Pisa, Italy; tarita.biver@unipi.it

**Keywords:** selectivity, G4 topology, porphyrin, phenanthroline, metal complex binding, pericyclic compounds

## Abstract

G-quadruplexes (G4) are now extensively recognised as a peculiar non-canonical DNA geometry that plays a prime importance role in processes of biological relevance whose number is increasing continuously. The same is true for the less-studied RNA G4 counterpart. G4s are stable structures; however, their geometrical parameters may be finely tuned not only by the presence of particular sequences of nucleotides but also by the salt content of the medium or by a small molecule that may act as a peculiar topology inducer. As far as the interest in G4s increases and our knowledge of these species deepens, researchers do not only verify the G4s binding by small molecules and the subsequent G4 stabilisation. The most innovative studies now aim to elucidate the mechanistic details of the interaction and the ability of a target species (drug) to bind only to a peculiar G4 geometry. In this focused review, we survey the advances in the studies of the binding of small molecules of medical interest to G4s, with particular attention to the ability of these species to bind differently (intercalation, lateral binding or sitting atop) to different G4 topologies (parallel, anti-parallel or hybrid structures). Some species, given the very high affinity with some peculiar G4 topology, can first bind to a less favourable geometry and then induce its conversion. This aspect is also considered.

## 1. Introduction

**Figure 1:**
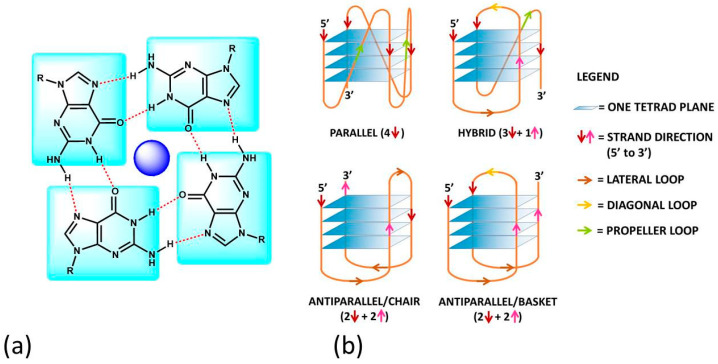
(**a**) The guanine-tetrad (G4) and (**b**) examples of structural conformations of G4s. In (**b**), the intramolecular type is taken into account; the same classifications hold in the intermolecular case. In (**b**), the oligonucleotide strand goes in the 5′ to 3′ direction; the vertical arrows (red or pink) indicate the direction of the part of the strand involved in G-tetrad formation, and the diagonal arrows refer to the direction of the loops. The latter can be of three types: (i) the lateral loop connects two adjacent guanines of the same G-quartet plane (brown arrow); (ii) the diagonal loop connects two opposite guanines of the same G-quartet plane (yellow arrow) and (iii) the propeller loop connects two guanines belonging to two different (upper and lower) G-quartet planes (green arrow).

A G-quadruplex (G4) is a four-stranded DNA or RNA structure with stacked guanine tetrads (G-tetrads) held together by eight hydrogen bonds (Figure 1a) [1]. At the end of the XX^th^ century, quadruplex sequences were identified at the end of telomeric DNA in eukaryotic chromosomes [2] and gene promoter regions [3]. These first findings constituted the start of enthusiastic studies on G4s, which became the target for cutting-edge research, in particular for the design and development of novel anticancer drugs. The studies on RNA G4s are more recent, but it is now clear that RNA G4s and G4s formed on RNA-DNA hybrids are also involved in regulating chromosome functions that involve DNA, RNA and RNA/DNA complexes [4,5,6,7]. In the last two decades, researchers searched for molecules that could selectively discriminate between G4s and double-stranded DNA/RNA polynucleotides. However, it quickly became clear that sequences that could fold into G4s could have produced species with a very rich structural polymorphism and that different G4s typologies could be associated with different cellular processes [8,9,10]. Selectivity is a key point for efficient therapies, so the problem moved to enable the differentiation of different types of G4s. Note that polymorphisms need to be carefully considered not only from a static point of view but also in the frame of several geometries connected by equilibria [11]. The major, dominating one will depend not only on geometrical/sequence factors but also on the medium (type and concentration of cations) and on the possible presence of some small molecule, specially designed to switch the reaction towards a peculiar geometry. This aspect, on the one hand, complicates much of the picture but, on the other hand, is fascinating and opens the way to new scientific strategies to reach the goal to find optimised drugs or even using G4 assemblies for obtaining responsive nanomaterials with electronic, biomedical and drug-delivery applications [12].

G4s can arise from the folding of a single strand (intramolecular G4). The formation of these quadruplex structures in human chromosomes is possible since the terminal nucleotides of telomeric DNA are single-stranded [13]. On the other hand, G4s can be formed by the combination of two or more separated strands (intermolecular G4s). G4s can display a wide variety of topologies (Figure 1b), as a consequence of several possible combinations of strand direction (intended as 5′ → 3′), number of G-tetrads, as well as variations in the size and the disposition of the loops (arising from the nucleotides not involved in the G-tetrads). G4s can be classified as parallel if the strands are all oriented in the same direction (4 ↑ or ↓) [14]. On the contrary, antiparallel structures derive from strands directed in different ways (2↑+ 2↓) [15]; these G4s can be arranged as a chair or basket-type structures depending on the loops’ position. If the structure is instead formed by three parallel and one antiparallel strand, the G4 is defined as a hybrid (3↑ + 1↓ or 3↓ + 1↑) [16]. 

The formation, stability and conformation of the quadruplexes are dependent on monovalent cations [17]. M^+^ ions can conveniently fit into the central channel of the G-tetrads, according to the strong negative electrostatic potential created by the guanine oxygen atoms [18]. The precise location of the cation depends on the nature of M^+^. Na^+^ ions have been found as centred in the plane of a G-tetrad as well as in the space between two successive G-tetrads; K^+^ ions are, instead, always equidistant between each G-tetrad plane and form a symmetric tetragonal bipyramidal configuration with the eight oxygen atoms [1]. Because K^+^ is much more abundant than Na^+^ in cellular environments, the knowledge of the human telomeric G4 structure in K^+^ solution is more important. K^+^ ions have been found to induce hybrid-type conformations for human telomeric G4, whereas Na^+^ ions tend to promote antiparallel structures [19]. M^2+^ ions may also play an important role [6]; for instance, Ca^2+^ will favour parallel structures [20]. 

**Figure 2:**
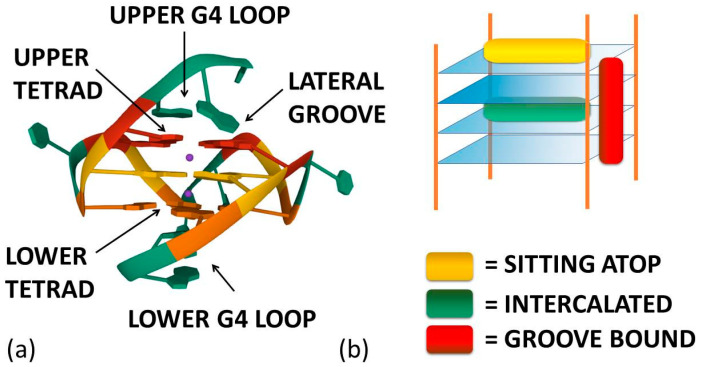
(**a**) Ligand–G4 interaction sites (the structure is PDB-1XAV, a monomeric parallel-stranded G4 from human c-MYC promoter). (**b**) Ligand–G4 complex types cartoon: the yellow rectangle refers to a species whose G4 interaction is driven by upper G4 stacking; the green one refers to a species able to intercalate itself between two adjacent tetrads; the red one refers to a molecule that chooses lateral grooves as the preferred binding site.

As already cited above, ligand–G4 interactions are extensively investigated since G4s are involved in important biological processes, especially concerning cancer cells [21]. Therefore, ligands that stabilize or induce the formation of G4 structures can be considered promising anticancer agents [22,23]. Besides crystals, as for geometries in solution, detailed CD and NMR spectroscopic studies, together with computational investigations [24,25,26,27,28], have provided a clear understanding of quadruplex structures. Additionally, innovative approaches such as 2D IR spectroscopy are proposed for analysing G4 structures [29]. On this basis, it is possible to develop a rational approach to the design of quadruplex binding ligands with potential anticancer activity [30]. Geometrical complementarity plays a major role in the search for high affinity and selectivity. Good G4 binders should present extended π-planar structures able to stack the external G-tetrads. Moreover, positively charged substituents promote the affinity with the grooves and the loops of G4 thanks to the electrostatic attraction with the negatively charged phosphate backbone. Lastly, a partial positive charge, substituting the cationic charge of the potassium or sodium that would normally occupy the G4 centre, can increase adduct stabilisation [31]. Optimal ligands should be selective for G4 over double-helix DNA since the double-stranded helix constitutes the major component of the human genome and its binding can prelude general cellular toxicity [32]. G4 selectivity can arise, at least in part, from the difference between the large, highly accessible surface area of a terminal quartet compared with the much smaller, less accessible G-C or A-T base pair surfaces of a typical duplex DNA [33]. Common G4 binders are macrocyclic ligands such as porphyrins, phthalocyanines and their metal complexes [34]. Meso-5,10,15,20-Tetrakis-(N-methyl-4-pyridyl)porphine (TMPyP4) represents the most commonly studied example for the porphyrin class [35]. Metal–salphen and metal–salen complexes have shown strong affinity and high selectivity for G4s as well [36]. Square-planar Ni(II)–salphen complexes have been found to inhibit telomerase activity by stabilizing G4 structures [37]. Aromatic molecules that bear charged peripheral chains (such as perylene diimide and phenanthroline derivatives) can bind G4 through the π-stacking interaction between the aromatic core and the G-tetrads along with electrostatic interactions between the substituents and the G4 backbone [38]. Investigations on non-planar metal complexes are rarer, but some examples have been reported in the literature anyway. For example, the binding properties of dinuclear [Ru(II)(phen)_2_(dppz)^2+^] complexes have been successfully investigated [39]. Concerning the binding position of the ligand, the intercalation of small molecules between the quadruplex tetrads is thought to be difficult because G4s are extremely stable and rigid. Besides, Figure 1a enlightens the presence of eight hydrogen bonds in one G-tetrad, i.e., more than the two or three hydrogen bonds present in each AT or GC base pairing, respectively. Accordingly, any possible G4 distortion requires a very high energy cost [40]. Thus, the stacking of the ligand on the outer surface of G4 as well as its disposition on the grooves appear to be more energetically favourable and probable binding modes (Figure 2) [8]. 

On the whole, this focused mini-review aims at helping the interested researcher to summarise recent findings on the binding features of small molecules to different G4 topologies, highlighting both the selectivity towards a peculiar G4 geometry and the ability to push this selectivity until the ability to switch the first-bound form into another preferred structure. To help to visualise the different behaviours, in the figures showing the molecular structure of the target compounds, red letters will evidence parallel G4s’ high selectivity, blue letters correspond to molecules selective for antiparallel topology, whereas the systems with other/mixed behaviour will have black lettering.

## 2. Selective Binders

### 2.1. Porphyrins

**Figure 3:**
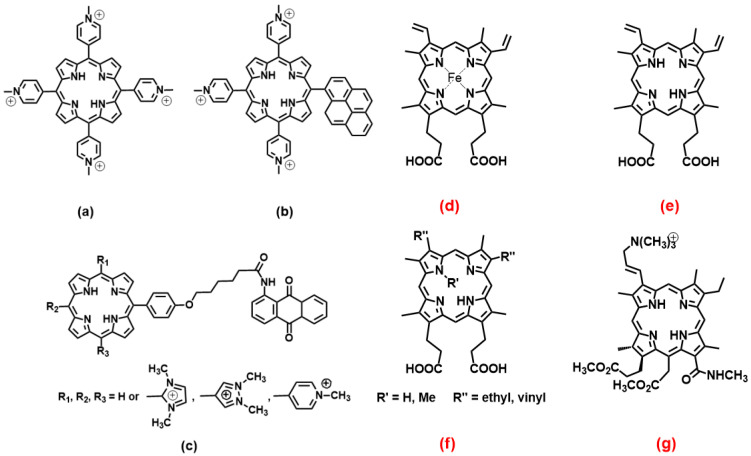
Molecular structures of the selective G4 binders of the porphyrin family reviewed here; (**a**) [42,43,44,45,46,48], (**b**) [49], (**c**) [50], (**d**) [51], (**e**) [52], (**f**) [53] and (**g**) [54]. In this and similar figures, **red** letters evidence **parallel** G4s high selectivity, **blue** letters correspond to molecules selective for **antiparallel** topology (absent here), and the systems with other/mixed behaviour have black lettering.

Porphyrin derivatives, although suffering from auto-aggregation, represent a family of molecules that comply with the geometrical request for broad, planar aromatic residues that could drive hydrophobic, π − π end-stacking with the outer G-tetrad planes of G4s. Besides, cationic porphyrins play an important role also in the frame of photosensitizers and are, also in that frame, already studied for medical applications [41]. As already cited, meso-5,10,15,20-Tetrakis-(N-methyl-4-pyridyl)porphine (TMPyP4, Figure 3a) is the golden standard for this class of molecules. Several studies have been performed on TMPyP4/G4 systems and have produced results that may appear controversial: some authors claim the absence of strong selectivity between different G4 forms and the binding or stabilisation of non-parallel topologies [42,43,44], and others enlighten the much higher affinity for parallel forms [45,46]. One point is the accessibility of the upper G4 tetrad. A basket-type antiparallel G4 or any form with diagonal loops will hinder the binding, whereas chair-type antiparallel G4s would still have high tetrad accessibility. A detailed discussion on this aspect is provided, for instance, by the work of Arora and Maiti (and references therein); in their study, they highlight the effect of loop orientation on TMPyP4/G4 (human telomeric) interactions and explain the preferential binding to parallel G4s over the antiparallel counterpart (one order of magnitude higher binding constants) [46]. Note that this type of study is based on oligos, which produce different topologies with different loops thanks to even small variations in the sequence [46]; in this light, loops’ length and type are strongly connected with (even single) nucleotide variations and it may likely be expected that a strong involvement of the loop in driving the binding features parallels a strong dependence of these features on the nucleotides forming the loop [47]. Docking studies, molecular dynamics simulations, calculations on hydrogen bonding, solvent-accessible surface area (SASA) and solvent-excluded surface area (SESA), along with the binding energies, unanimously supported the better binding of TMPyP4 with the parallel G4 [45]. However, not all systems follow this rationale. In addition to end-stacking, TMPyP4 can show also a high affinity for G4s’ grooves, and this reduces the selectivity among parallel/antiparallel/hybrid structures that are all efficiently bound [42]. Additionally, it was found that TMPyP4 unselectively binds to a thrombin-binding aptamer forming parallel and anti-parallel basket-type G4s but with a binding mode outside of the quadruplexes and based only on weak electrostatic interactions with the phosphates [48]. 

As for similar systems, a pyrene derivative of TMPyP4 (Figure 3b) was shown to bind (and destabilise) similarly parallel and antiparallel G4s, but the latter was chair-type; these authors evidenced the importance of the length (number of T residues) in the lateral loop to modulate the binding [49]. Theoretical studies on antraquinone derivatives (Figure 3c) bearing also methyl imidazole or methyl pyrene have produced information on the details of the binding, which drive the preference to bind parallel or mixed hybrid structures compared to the antiparallel structure [50]; besides other thermodynamic details, this work enlightens the role played by “tail” substituents, which produce an additional effect to the sitting-atop stacking of the porphyrin core in loop binding. 

Moving from the “TMPyP4-like” species, another important porphyrin class is that of heme. The heme iron complex itself (Figure 3d) was found to very selectively bind to the 3′-terminal of dimeric parallel G4s, whereas no binding occurred for the dimeric antiparallel counterpart [51]. The results are discussed also in light of the importance of the orientation of the ring oxygen atoms from the guanine deoxyribose moieties to the G4 plane for the stacking of π-conjugated macrocycles such as heme; conversely, the binding is not largely affected by the negative electrostatic potential due to nearly phosphate groups. In the absence of the iron metal centre (turning heme to protoporphyrin IX, PPIX, Figure 3e), the selectivity is maintained: PPIX binds parallel G4s 100-fold better than antiparallel ones so that PPIX can even be used as a sensitive turn-on sensor for parallel G4s and potassium ions (as parallel forms inducers) [52]. Again, the selectivity is maintained also if the porphyrin core is distorted and hindered by N-methylation, such as in the non-planar derivative N-methyl mesoporphyrin IX (Figure 3f, R’ = Me, R’’ = ethyl) [53]. The work in the direction to obtain the optimum discrimination between peculiar G4s geometries should not crash with the desired selectivity over double-stranded polynucleotides. It was found that the porphyrin shown in Figure 3g, among other similar photosensitizers, is the better G4 stabiliser over duplex DNA (with significant improvement concerning TMPyP4, which is also a natural DNA binder) and also showed a marked preference for sitting-atop binding to the 3′-end of parallel G4s over antiparallel ones; the affinity is the highest for G4 from KRAS proto-oncogene promoter, a target for cancer therapy [54]. 

### 2.2. Phenanthrolines and Their Metal Complexes

**Figure 4:**
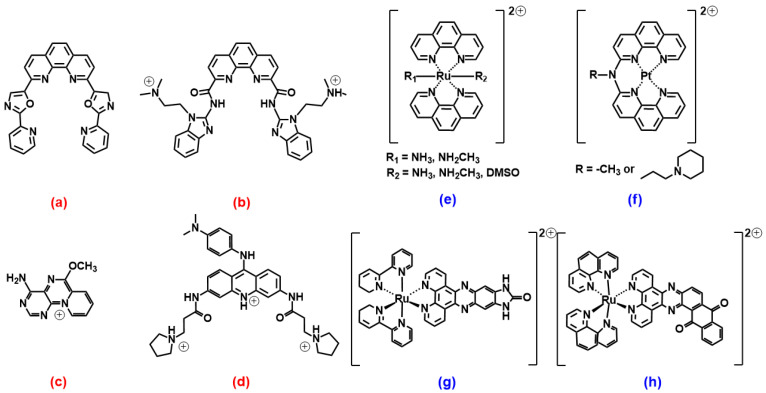
Molecular structures of the selective G4 binders of the phenanthroline family reviewed here: (**a**) [55], (**b**) [57], (**c**) [58], (**d**) [59], (**e**) [63], (**f**) [64], (**g**) [65] and (**h**) [66]. **Red** letters evidence **parallel** G4s’ high selectivity, **blue** letters correspond to molecules selective for **antiparallel** topology, and the systems with other/mixed behaviour have black lettering (absent here).

**Figure 5:**
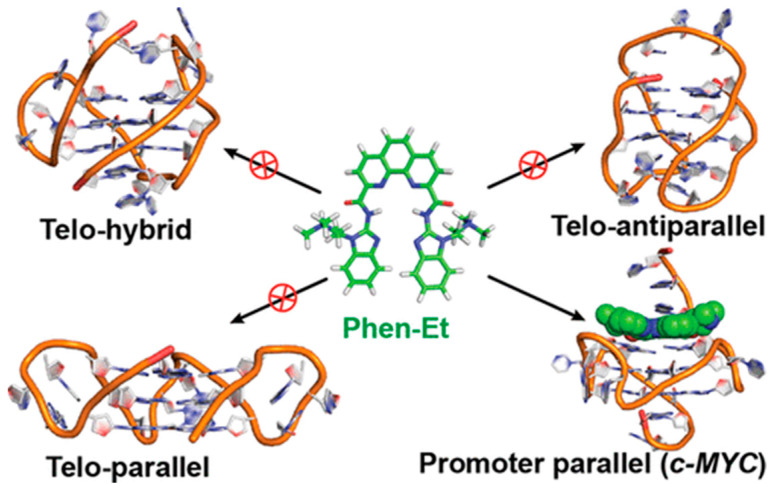
Selectivity of a bisbenzimidazole carboxamide phenanthroline derivative for a peculiar parallel G4 topology [57]. Reprinted with permission from ACS Chem. Biol. 2015, 10, 3, 821–833. Copyright 2014 American Chemical Society.

Another golden standard among families widely studied as themselves or as ligands for metal ions are 1,10 phenanthroline derivatives. A phenanthroline-bis-oxazole (2,9-bis(2-(pyridine-2-yl)oxazole-5-yl)-1,10-phenanthroline, Figure 4a) was found to stabilise much more c-MYC (an oncogene promoter with parallel topology) over antiparallel and hybrid G4 topologies, and with very high selectivity over duplex structures; it is a tight and specific binding, where the molecule end-stacks at both ends of the G4 [55]. In this architecture, the phenanthroline derivative mimics oligo-heteroaryle species (see below), which were one of the first species highlighted to efficiently discriminate between G4 topologies [56]. A bisbenzimidazole carboxamide derivative (Figure 4b) was found, again, to be the best species among other similar derivatives to selectively bind parallel forms [57]; however, interestingly, it was able also to discriminate between two types of parallel forms (Figure 5). In their work, the authors conduct several spectroscopic experiments and molecular modelling and dynamics calculations and highlight the importance of the length of the lateral residues and of the presence of flanking residues from the G4 to gain specificity of the binding features.

Other species (Figure 4c,d), which somehow resemble the previously cited ones, also showed selective binding towards parallel G4 forms [58,59]. As for 4c, the result is discussed based on the usual end-stacking favoured by the presence/absence of hindering by the loops, with the addition of some interesting analysis on fluorescence lifetimes and by the careful check of the absence of positive induced dichroic signals (ICD), taken as an indicator of groove binding to G4 [58]. On the other hand, Figure 4d reports the phenylaminoacridine also known as BRACO19, which constitutes a golden reference for G4 binders, to such an extent that it can be used as a fluorescent tag for exchange experiments to prove G4 binding by the competitor [60]. The paper above cited on BRACO19/G4 interaction [59] uses molecular dynamics including explicit solvents to improve a previous theoretical work [61]: both papers emphasise the higher affinity for a G-quartet stacked binding mode. These data agree with a previous work [62] that afforded the crystal structure of the BRACO19/G4 adduct, showing the BRACO19 molecule stacking directly onto the 3′ end G-tetrad face.

What seems to happen more often is that, in the presence of selectivity, this would quite often be in favour of the parallel topology. The recognition will be based on the accessibility of the end-tetrads, which are free of the shielding effects of some diagonal, or even lateral, loops. However, metal complexes of phenanthroline ligands are species out of this trend. Octahedral ruthenium complexes (Figure 4e) were specially designed to hinder any double-strand DNA intercalation thanks to the *trans* coordination positions of the *z*-axis; in addition to this excellent selectivity, these metal complexes were found to interact preferentially with basket-type antiparallel G4s [63]. This occurs in particular in the presence of NH_3_ residues, which can align with the cation cavity and strongly interact with the G4 via H-bonds, with a geometry that is optimal only in the case of basket-antiparallel G4. Similar platinum compounds (Figure 4f) also show very interesting peculiar features: they stabilise both DNA and human telomeric RNA (*TERRA*) G4 sequences and show a preference for basket-type antiparallel topology [64]. The authors emphasize the importance of the presence of the piperidine residue to both discourage interaction with double-stranded polynucleotides and improve, through interaction with the lateral loop, the recognition of the antiparallel form. 

Studies on ruthenium complexes were performed also on the family of the very well-known [Ru(bpy)_2_(dppz)]^2+^ species (bpy = 2,2′-bipyridine, dppz = dipyrido[3,2-a:2′,3′-c]phenazine); the aim was also to investigate and highlight the possible differences in the binding features of the different metal complexes enantiomers [65]. It was found that, if [Ru(bpy)_2_(dppz)]^2+^ itself does not show striking discrimination features, the imidazolone derivative (Figure 4g) shows selective binding, with a preference for antiparallel basket structures and a stronger affinity of the Λ-enantiomer over the Δ-enantiomer. Interestingly, this affinity scale is also opposite to what generally occurs for double-stranded polynucleotides, where Δ-enantiomer > Λ-enantiomer.

A recent work by McQuaid and co-workers [66] confirms this trend: they obtained the crystal of the adduct between a G4 antiparallel (chair) topology and the ruthenium complex Λ-[Ru(phen)_2_(qdppz)]^2+^ (Figure 4h). Their data confirm the presence of an enantiospecific 1:1 G4 binding to this peculiar geometry, with the metal complex positioning in an intercalation upper cavity created by a terminal G-quartet and a lateral loop.

### 2.3. Coumarin, Quercetin, Berberine and Pericyclic Compounds

**Figure 6:**
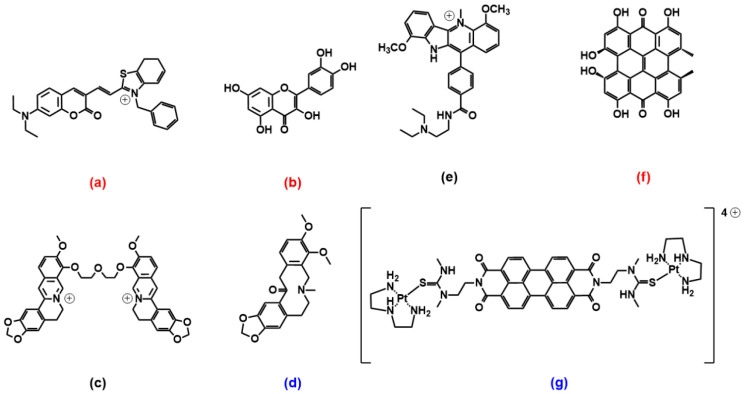
Molecular structures of the selective G4 binders of the pericyclic compounds family reviewed here: (**a**) [67], (**b**) [68], (**c**) [69], (**d**) [70], (**e**) [71], (**f**) [72] and (**g**) [73]. **Red** letters evidence **parallel** G4s high selectivity, **blue** letters correspond to molecules selective for **antiparallel** topology, and the systems with other/mixed behaviour have black lettering.

A coumarin–benzothiazole hybrid (Figure 6a) was recently found to exhibit enhanced fluorescence emission upon sitting-atop binding to a parallel G4 topology, with no response for the antiparallel counterpart; this “turn-on” response is used to sense the presence of parallel-form inducers such as K^+^ ions, with excellent selectivity for K^+^ in the presence of other potential metal ion interferences [67]. An interesting work by Saxena and co-workers compares the energies for the binding of the flavonoid quercetin (Figure 6b) with some DNA and RNA G4s: differently from the more abundant sitting-atop binding mode, quercetin interacts in all cases from the G4 groove and is found to produce adducts that are more stabilised in the case of parallel DNA G4, whereas RNA G4 adducts are all less favoured (likely due to less efficient hydrogen bonding) [68]. Berberine-like structures also showed important potentialities. The berberine dimer shown in Figure 6c (only this peculiar structure with the shortest polyether linker among other linkers tested) showed very high selectivity towards mixed-type multimeric G4 over parallel or antiparallel monomeric G4s [69]. External binding and end-stacking modes were found to apply, whereas intercalation was excluded. In the shortest linker, the distance between the two berberine subunits optimally matches the distance from the centre of one G4 plane to the centre of another one in mixed-type G4; thus, the compound is likely to simultaneously interact with the two G4 subunits. The adduct is also stabilised by a strong contact with two propeller loops. The natural alkaloid allocryptopine (Figure 6d) showed a binding mode via groove and/or loop binding to G4s that is distinct from the conventional π-stacking of the ligands on external G-quartets and drove selectivity for antiparallel topologies; in this paper, the binding details and geometries are further inspected using steady-state and time-resolved anisotropy measurements [70].

An indoloquinoline derivative (Figure 6e) can discriminate among different G4 topologies, with a preference for parallel-stranded MYC quadruplexes but also for a hybrid [71]; its crescent-shaped and non-coplanar arrangement structure has limited stacking interactions with respect to other species, but the side chain provides better discrimination between different forms [71].

As for pericyclic compounds with extended planar aromatic cores, a couple of interesting examples are provided by the two species shown in Figure 6f,g. Hypericin (Figure 6f), an anthraquinone plant extract, is found to selectively recognise parallel G4s by end-stacking; here, despite the extended planarity, some balance between the core and the peripheral hindering functionalities enables also achieving discrimination over double-stranded DNA into which no intercalation is found to occur [72]. On the other hand, the perylene bisimide platinum complex in Figure 6g, despite a central part that, in principle, could again drive towards sitting-atop parallel G4s discrimination, selectively recognises antiparallel topologies, even if the binding mode is still end-stacking with 2:1 stoichiometry [73]. Interestingly, the same paper also compares the results obtained between the antiparallel DNA-G4 and the RNA-G4 analogue and highlights the details of a binding mechanism that differs from DNA-G4 and where a double-layer of ligands is sandwiched between two RNA-G4s. In our group, we also studied, by a combined spectroscopic and theoretical approach, the binding of a water-soluble perylene diimide to G4s. Molecular dynamics confirmed the formation of H-bonds and very favourable complementary geometry for lateral/groove binding, but the same is not found in sitting atop structures; on the whole, both experiments and calculations show that the two binding modes are possible and may be simultaneously present [74].

### 2.4. Miscellaneous

**Figure 7:**
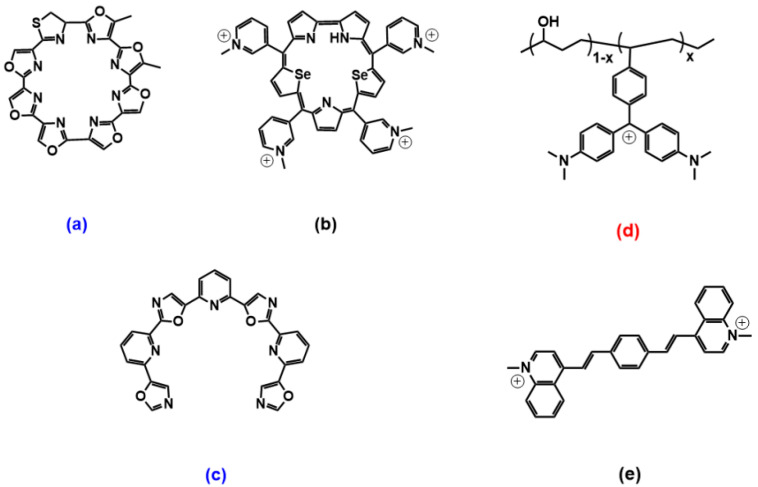
Molecular structures of the other selective G4 binders reviewed here: (**a**) [79], (**b**) [79,80], (**c**) [56], (**d**) [82] and (**e**) [83]. **Red** letters evidence **parallel** G4s’ high selectivity, **blue** letters correspond to molecules selective for **antiparallel** topology, and the systems with other/mixed behaviour have black lettering.

**Figure 8:**
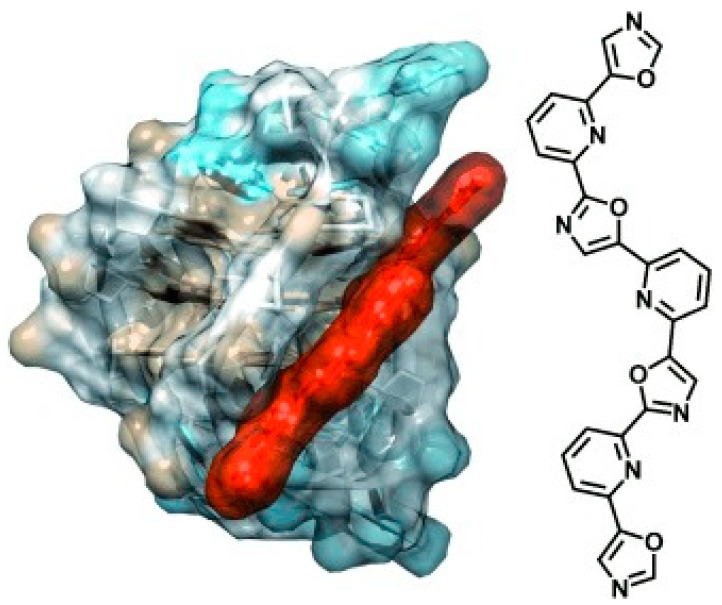
The unique geometrical features of TOxaPY enable the recognition of a peculiar antiparallel topology by groove-binding to it. Reprinted with permission from Wiley—Ref. [56].

Many other papers cite the ability of the more different species to discriminate between G4′s forms. As for examples of parallel (c-MYC being a golden standard) G4 discrimination, we may cite benzothiazole [75], indolyl-quinolinium scaffolds [76] and pyrimidine/purine [77,78] derivatives. Telomestatin and sapphyrin cyclic heteroaryles (Figure 7a,b) deserve peculiar attention as one of the first species shown to selectively bind to a peculiar G4 (antiparallel basket-type for Figure 7a, hybrid for Figure 7b) [79]. Sapphyrin, additionally, strongly and selectively binds to a mixed parallel/antiparallel G4, which is the form obtained by the switch it causes on a parallel c-MYC topology [80]; also, again, sapphyrin can convert antiparallel basket G4s into hybrids [79]. More recent studies on telomestatin acyclic counterpart (TOxaPy, Figure 7c) demonstrated that the unique geometrical features of this probe let it interact very selectively only with the groove of an antiparallel G4 coming from a peculiar sequence, differentiating it from another antiparallel G4 (Figure 8) [56]. Another, totally different class of molecules that may deserve some comment is that of triphenylcarbenium derivatives, such as triarylmethane dyes. Crystal violet is not so selective (only a slight preference for antiparallel forms) [81], but malachite green, linked to a poly(vinyl alcohol)/polyethylene copolymer (Figure 7d), showed a preference for parallel G4s over mixed parallel/antiparallel structures according to a binding that is absent in the dark but can be photoinduced [82]. The new perspective of turning on the binding was inspected also because of the possible toxicity of the dye probe due to cyanide and radicals production; however, the process was found to be non-cytotoxic, probably thanks to the presence of poly(vinyl alcohol) hydrophilic polymer. Arylstilbazolium species are other photoisomerizable compounds that showed interesting light/geometry dependent binding features: bis(vinylquinolinium)benzene (Figure 7e) could be switched by light between E,E and E,Z isomers, with E,E favourably interacting with antiparallel basket-type G4s and E,Z preferring parallel propeller-type c-MYC [83].

## 3. Topology Switch Inducers

Several molecules, belonging to several families, were found to stabilise G4 forms to such an extent to induce the folding of oligonucleotide sequences to G4 architectures, also in the absence of cations such as K^+^ or Na^+^. Just as in the examples, we may cite here the cyclic heteroaryle telomestatin (Figure 7a) [79], thioflavin T [84,85], the perylenetetracarboxylic diimide PIPER [86], and metal complexes such as [Ru(bpy)_2_(dppz)]^2+^ [87] and alkynylplatinum(II) terpyridine complexes (these latter ones showing an interesting groove-binding mode) [88]. However, we will review here only some species able to let the G4 switch from one topology to another in a medium containing stabilising cations. A very good recent paper by Ma and co-workers reviews in detail G4 ligands that induce conversion between different topologies [10]. However, we will produce here below some additional/alternative information.

### 3.1. Porphyrins

**Figure 9:**
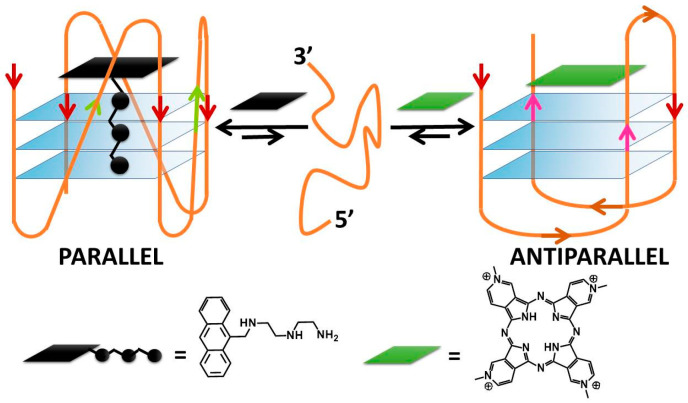
DNA conformational switching according to Rodriguez and co-workers [90].

TMpyP4 can bind to hybrid structures, both by the external G-tetrad planes and with the bases of the interconnecting loops; this interaction produces a destabilisation of the hybrid geometry and finally leads to a switch to basket-type antiparallel topologies. However, this occurs under diluted conditions only; the same authors analysed what happens under molecular crowding conditions (40% *w*/*v* of PEG200) and found that the parallel structure becomes the majority and is not affected anymore by TMPyP4 binding [89]. Methyl mesoporphyrin IX (Figure 3f) increases the parallel component of different G4s [53]. A peculiar behaviour, already evidenced in Ma’s paper, deserves to be highlighted also here: porphyrazine can induce antiparallel quadruplex conformations and can be used also along with a complementary ligand to controllably switch quadruplex conformations between parallel and antiparallel topologies (Figure 9) [90]. Note that even more complicated systems can be engineered: N-methyl mesoporphyrin IX (NMM) was utilized to first convert a hybrid G4 into the parallel fold, and then cationic hemicyanine dyes are used to exchange NMM and favour antiparallel or hybrid topologies. Thus, NMM affords a topology that differs from one directly favoured by the hemicyanines and enables the monitoring of the binding by the loss of its fluorescence upon binding [91]. The planar square-based Cu(II) 12-MC-4 metallacrown has some geometrical similarities with porphyrin metal complexes and, similarly to what happens, for instance, in the case of the iron-porphyrin hemin [92,93], induces transformations into the parallel form [94].

### 3.2. Phenanthrolines Derivatives and Their Metal Complexes

**Figure 10:**
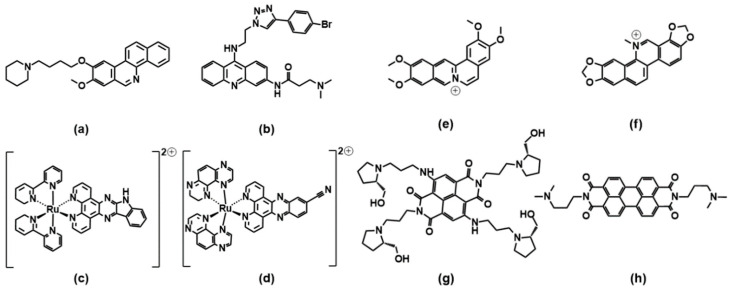
Molecular structures of the G4-topology switchers reviewed here; (**a**) [95], (**b**) [9], (**c**) [96], (**d**) [98],(**e**) [99], (**f**) [100], (**g**) [101], (**h**) [102].

**Figure 11:**
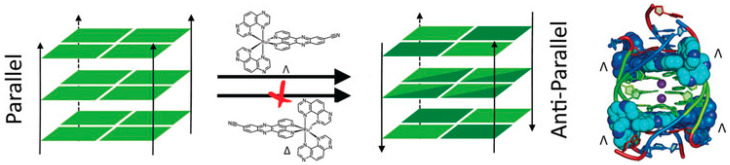
Drawing summarising the enantiospecific ability of a Ru–dipyridophenazine derivative to drive a parallel–antiparallel G4′s transition. Reprinted with permission from Wiley—Ref [98].

A benzophenanthridine derivative (Figure 10a) can switch antiparallel G4s into parallel ones [95]; interestingly, among other similar derivatives, it is the only molecule able to produce this effect on G4 sequences from HIF1α (the subunit of a hypoxia-inducible factor heterodimeric protein) and to consequently interfere with HIF1α binding to a transcriptional factor. The same switching behaviour from antiparallel to parallel is also found for a 9-aminoacridine derivative (Figure 10b) on a G4-forming sequence, which is the one identified in the promoter region of Nrf2 (nuclear factor (erythroid-derived 2)-like 2) [9]. As for metal complexes, ruthenium ones are the more abundantly studied. The Ru–indoloquinoline complex shown in Figure 10c can convert a hybrid telomeric structure into an antiparallel G4 and also destroy the G4 structure in the case of interaction with parallel c-MYC [96]. The hybrid-antiparallel conversion ability is retained when a –OCH_3_ functionality is added to the indoloquinoline ligand [97]. In this family of ruthenium complexes, the presence of chirality is always an interesting aspect to take into account. The –CN derivative shown in Figure 10d showed a marked enantiospecific behaviour as Λ-[Ru(TAP)_2_(11-CN-dppz)]^2+^ (TAP = 1,4,5,8-tetraazaphenanthrene, dppz = dipyridophenazine) changes parallel topologies into antiparallel ones, whereas Δ-[Ru(TAP)_2_(11-CN-dppz)]^2+^ is not a switch inducer (Figure 11). This behaviour is analysed in the details thanks to the comparison between crystallographic data, synchrotron radiation circular dichroism (SRCD) and spectroscopic/melting tests, elucidating that the observed behaviour is a consequence of the increased stabilisation of syn-guanosine by the Λ-enantiomer [98].

### 3.3. Pericyclic Compounds

Coralyne (Figure 10e), a known synthetic protoberberine alkaloid, can induce the transformation of hybrid/antiparallel G4 to parallel forms. Detailed circular dichroism, molecular dynamics and ^1^H/^13^C/^31^P NMR analyses produced a picture with coralyne stacking at two upper and lower tetrads, accompanied by the formation of a hydrogen bond; i.e., a highly specific interaction at the molecular level that induces the specific changes in the G4 backbone, which may be the basis for the switch [99]. Similarly, the sanguinarine alkaloid (Figure 10f) can induce the conformational change from hybrid G4 to antiparallel basket-type conformations [100].

The naphthalene diimide (NDI) shown in Figure 10g can induce a switch of G4 morphology from hybrid to parallel; the authors use their spectroscopic data (circular dichroism, NMR and fluorescence tests, including Förster resonance energy transfer experiments) to gain information on a detailed step-by-step reaction mechanism for which they propose the intermediates’ geometries [101]. More extended perylene diimides (PDI) also interfere with the G4 topologies: the molecule shown in Figure 10h is found, in particular by electrospray ionization mass spectrometry, to drive the formation of a parallel G4 form starting from a mixed parallel/antiparallel geometry [102].

## 4. Conclusions

In this focused mini-review are summarised systems able either to specifically interact only with peculiar G4 topologies and/or to induce a switch between different forms. It can be noted that the studies on RNA-G4s are much less abundant. RNA-G4s are supposed to have less polymorphism, preferring parallel topologies (even if an antiparallel RNA-G4 was found for human telomere RNA, [103]) and are generally more rigid and stable than their DNA-G4 counterpart [104]. These characteristics, other than hindering efficient host–guest matching, can offer the basis of RNA/DNA selective recognition. Some small molecules, indeed, bind RNA-G4 significantly better than the DNA-G4 competitor (for instance TMPyP4 [105] and chelerythrine alkaloid [106]). The second remark is on the wide variety of compounds and geometrical structures that may be active. Under these circumstances, the final result appears to be an intricate superimposition of interactions with the tetrads and the loops, whose relative strength comes from small details in both host and guest structures. The parallel geometry offers more accessible upper/lower tetrads, and it is often the preferred (and selected) topology and also that which results from the switches starting from less stable forms. However, the binding details are rarely the same, and many exceptions to this simple rule exist. For instance, in addition to the many other examples reviewed here, the presence of a planar extended aromatic residue is not sufficient to ensure some sitting-atop binding on the G4 upper/lower tetrad [107]. The inspection of the literature enlightens a picture where each system makes its own story, as each of the molecules has a reactivity that is the sum of subtle details that are hard to pre-evaluate. This means that there is still much room for studies aimed at finding the key compound to exactly match a peculiar G4 geometry involved in some interesting medical pathway. Additionally, a recent paper from Vianney and Weisz [108] evidences how a lateral loop can turn into a quadruplex–duplex junction, which is increasingly considered a promising target for technological and medicinal applications and, consequently, for which there is a need for specific binders. Additionally, the role of the loop and/or of “tails” substituents to better and more efficiently tune selectivity with respect to G-tetrad stacking-only focused species is evidenced by the here-cited papers. In silico screening of a large number of compounds may help select small molecules with optimal characteristics, such as hydrogen-bond acceptor/donor groups or lateral substituents perfectly matching to lateral grooves/hydrophobic pockets (whilst keeping high selectivity with respect to double-stranded polynucleotides). However, theoretical calculations would need the verification by the experimental counterpart, and a particular role is played in this direction by crystal structures which are, on the other hand, not always available. Many studies do not go too much into the mechanistic details. The complete thermodynamic profiles for quadruplex–ligand interactions are limitedly offered, and a rigorous assessment of the relationship between the structure and binding mode is not always available nor easy to achieve (see for instance [109] where, also, an interesting study on salt dependence is provided). In this frame, the kinetic studies, which are even less abundant, may play a role. Kinetic studies could be precious to enlighten the details of some geometrical rearrangement [110]. Note that some papers often emphasise selectivity rules only based on binding constants calculations. NECTAR—COST Action 18202 coordinates the expertise of a huge network of researchers involved in binding constants evaluation. The NECTAR work shows the dramatic repercussions on the final numbers evaluations of the type of data analysis (equation/software) used. The same work is evidencing the need for very careful data collection, bias sources evaluation, and strict protocols (including buffers) to improve the robustness of these numbers. This critical homogenisation process is fundamental for data comparison not only intra-laboratory but also inter-laboratory, to enable a merging of the huge number of results produced by the community that would enable extracting reliable structure–activity relationships (SARs). It would be important for researchers to converge over an experimental protocol and a reaction medium which, given that the G4 geometries are highly dependent on the type/concentration of ions, should be also optimized for future use in vivo.

## Data Availability

Not applicable.
